# NMR Metabolomic Profiling of Differentiated SH-SY5Y Neuronal Cells: Amyloid-β Toxicity and Protective Effects of Galantamine and Lycorine

**DOI:** 10.3390/cells14070525

**Published:** 2025-04-01

**Authors:** Arian Kola, Filippo Costanti, Jordan Kahfi, Abdul-Hamid Emwas, Mariusz Jaremko, Daniela Valensin

**Affiliations:** 1 Department of Biotechnology, Chemistry and Pharmacy, University of Siena, Via Aldo Moro 2, 53100 Siena, Italy; arian.kola@unisi.it; 2 Department of Information Engineering and Mathematics, University of Siena, 53100 Siena, Italy; costanti@student.unisi.it; 3 Division of Biological and Environmental Sciences and Engineering (BESE), King Abdullah University of Science and Technology (KAUST), Thuwal 23955-6900, Saudi Arabia; jordan.kahfi@kaust.edu.sa (J.K.); mariusz.jaremko@kaust.edu.sa (M.J.); 4 KAUST Core Laboratories, King Abdullah University of Science and Technology, Thuwal 23955-6900, Saudi Arabia; abdelhamid.emwas@kaust.edu.sa; 5 Consorzio Interuniversitario Risonanze Magnetiche di Metalloproteine (CIRMMP), Via L. Sacconi 6, 50019 Firenze, Italy

**Keywords:** galantamine, lycorine, SH-SY5Y, amyloid-β, Alzheimer’s disease, alkaloids, oxidative stress, glutamate–glutamine cycle, pyruvate, metabolomics

## Abstract

Alzheimer’s disease (AD) is a multifactorial neurodegenerative disorder characterized by metabolic dysregulation, oxidative stress, amyloid-β (Aβ) aggregation, metal dyshomeostasis, and mitochondrial dysfunction. Current treatments provide only symptomatic relief, highlighting the need for novel therapeutic strategies. This study investigates the metabolic effects of the alkaloids galantamine (GAL) and lycorine (LYC) in differentiated SH-SY5Y neuroblastoma cells, an established in vitro model for AD, which acquire a neuronal phenotype upon differentiation. Using untargeted and targeted NMR-based metabolomics combined with multivariate statistical analysis, we analyzed extracellular metabolic profiles under basal conditions and following Aβ42 exposure, both in the presence and absence of GAL and LYC. Our findings reveal distinct metabolic responses to Aβ toxicity, with significant alterations in pyruvate and glutamine metabolism. Both GAL and LYC contributed to the restoration of glutamine and lysine homeostasis, but LYC had a more pronounced effect, better sustaining cellular energy balance and mitochondrial function. Unlike LYC, GAL treatment was associated with pyruvate accumulation, highlighting a distinct metabolic response between the two compounds. These variations may reflect distinct mechanisms of action, potentially influencing their therapeutic roles in counteracting Aβ-induced toxicity. This study highlights the value of metabolic profiling for assessing neuroprotective agents and reinforces the potential of natural alkaloids in this context.

## 1. Introduction

Alzheimer’s Disease (AD) is a progressive neurodegenerative disorder and the most common cause of dementia, affecting over 55 million people globally, with nearly 10 million new cases reported annually. Despite being identified over a century ago, no cure is currently available, and its prevalence continues to rise, largely due to increased life expectancy. This prolonged lifespan exposes individuals to heightened oxidative stress, disrupting neuronal balance and accelerating disease onset.

AD is widely recognized as a multifactorial disorder, with interconnected pathological mechanisms including Aβ aggregation, tau hyperphosphorylation, oxidative stress, metal ion dyshomeostasis, and neuroinflammation [1,2]. These processes interact to form a complex network that drives neuronal dysfunction and cognitive decline, making the disease particularly challenging to treat [3]. The amyloid cascade hypothesis, a leading explanation for AD pathology, posits that the abnormal accumulation of Aβ peptides initiates a cascade of events leading to neurotoxicity, synaptic loss, and eventual brain atrophy [4].

Currently, the treatments for AD are limited to symptom management rather than addressing the underlying causes. These include β- and γ-secretase inhibitors to reduce Aβ formation, acetylcholinesterase (AChE) inhibitors to enhance cholinergic signaling, anti-inflammatory drugs to mitigate neuroinflammation, and antioxidants to neutralize free radicals [3,5,6]. However, these interventions offer only modest benefits and do not halt disease progression. The recent approval of Aducanumab and Lecanemab, two anti-Aβ antibodies, by the FDA represents a milestone in AD therapy [7,8]. Despite this, to further advance treatment, it is essential to deepen our understanding of the cellular and molecular mechanisms driving AD [9], together with the identification of reliable biomarkers, which are crucial for early-stage detection of AD, enabling timely intervention [10].

**Figure 1:**
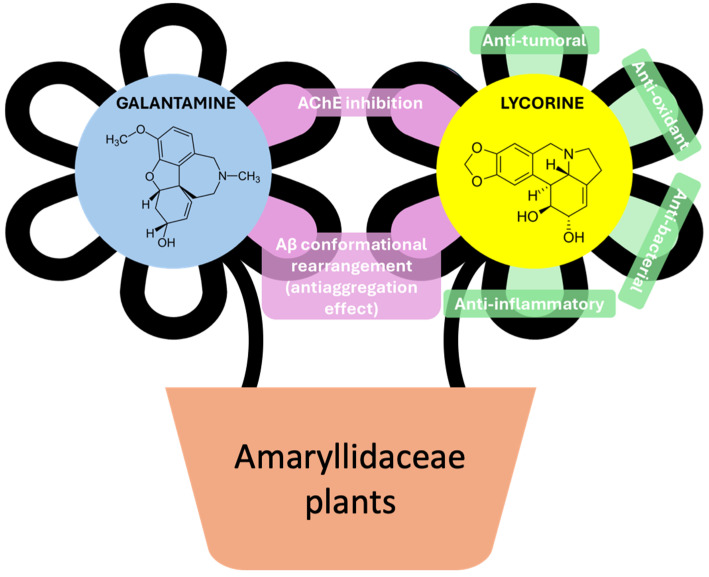
Chemical structures and biological activity of galantamine (GAL) and lycorine (LYC).

Ongoing research focuses on identifying new therapeutic strategies aimed at addressing the multifactorial nature of AD. Efforts include the development of molecules that inhibit Aβ aggregation, modulate tau pathology, and restore metal ion homeostasis. Additionally, a deeper understanding of pathogenic protein conformational changes and their role in neurodegeneration continues to guide the search for novel treatments that target the root causes of AD. Over the last several years, AD’s multifactorial nature has driven the exploration of natural bioactive compounds as potential therapeutic agents. Derived from plants and other natural sources, these compounds, such as polyphenols, alkaloids, vitamins, and terpenoids, offer diverse chemical properties and biological activities that make them valuable tools for modulating neurodegenerative processes [11,12,13]. Natural compounds, such as polyphenols, are widely recognized for their potent antioxidant properties. Molecules like rosmarinic acid, curcumin, and resveratrol effectively neutralize reactive oxygen species (ROS), thereby protecting neuronal cells from oxidative damage in vitro [14,15,16]. The alkaloid galantamine, commonly used in AD therapy, functions as an AChE inhibitor, increasing acetylcholine levels and enhancing cholinergic neurotransmission in AD patients [17]. Additionally, several natural compounds exhibit anti-aggregation properties, interacting directly with misfolded proteins to stabilize non-toxic conformations or prevent aggregation [18]. Among these, galantamine (GAL) and lycorine (LYC) are two alkaloids derived from the *Amaryllidaceae* family (Figure 1) that have demonstrated potential in mitigating amyloid-beta (Aβ) toxicity [19,20]. LYC has been extensively studied for its antiviral, anti-inflammatory, and antitumor properties as well [21]. Recent investigations have explored its neuroprotective potential against Aβ-induced toxicity. A comparative study demonstrated that LYC interacts with Aβ peptides, inhibiting their aggregation and reducing the associated neurotoxicity in vitro, offering protective effects similar to those of GAL [20]. Although both alkaloids interact with Aβ, promoting a conformational rearrangement, the cellular mechanisms underlying their neuroprotective activity remain to be clarified.

Cellular metabolomics is a critical tool for understanding therapeutic treatments, particularly for complex diseases like AD. It provides detailed insights into metabolic disruptions and cellular responses to therapies, revealing both efficacy and potential side effects. A robust NMR metabolomics method was developed using SH-SY5Y neuroblastoma cells to investigate the cellular effects of sub-toxic levels of alpha-synuclein and amyloid-beta (Aβ40 and Aβ42) in their monomeric, oligomeric, and fibrillar forms. The study identified eight key metabolites altered by protein aggregation, highlighting disruptions in lipid metabolism, neurotransmission, and oxidative stress adaptation [22]. Recent metabolomic studies using SH-SY5Y neuroblastoma cells have revealed that liraglutide, a novel antidiabetic drug with neuroprotective effects against neurodegenerative diseases, modulates energy metabolism in SH-SY5Y cells by promoting gluconeogenesis, enhancing oxidative phosphorylation, and suppressing glycolysis [23]. Similarly, the neuroprotective effects of *Dunaliella salina* extract in SH-SY5Y cells were linked to lipidomic alterations, suggesting a significant role for carotenoids and other minor compounds in modulating cell membrane properties and function [24].

In this study, the extracellular metabolic profile of differentiated SH-SY5Y cells was investigated by combining NMR spectroscopy and chemometric analysis. This approach provided a detailed overview of the metabolome under basal conditions and in response to Aβ exposure, both in isolation and in the simultaneous presence of GAL and LYC. By comparing these profiles, the study aimed to identify the biological pathways influenced by the two compounds and their potential roles in counteracting the metabolic disruptions induced by Aβ toxicity. The analysis focused on the key metabolic pathways such as energy metabolism, oxidative stress responses, and neurotransmitter biosynthesis, providing insights into how GAL and LYC may modulate the cellular processes associated with AD. The findings were then correlated with previously conducted cellular studies, which assessed the neuroprotective, antioxidant, and anti-aggregation properties of these alkaloids, which are known to weakly interact with Aβ and mitigate its cellular toxicity in vitro [20]. By integrating metabolomic data with cellular-level observations, this study offers useful insights into the ways GAL and LYC may help mitigate Aβ-induced toxicity, highlighting their role in Alzheimer’s disease therapy.

## 2. Materials and Methods

### 2.1. Materials

B-27^TM^ Supplement (50×), serum free, Neurobasal^TM^ Plus Medium, and GlutaMAX^TM^ were supplied by Thermo Fisher Scientific (Waltham, MA, USA). Galantamine hydrobromide from *Lycoris* sp. (≥94% HPLC) and Lycorine hydrochloride (≥98% HPLC) were purchased from Sigma-Aldrich (Schnelldorf, Germany). SH-SY5Y neuroblast cells were purchased by Merck KGaA (Darmstadt, Germany). Human Aβ42 was supplied by GenScript Biotech (Rijswijk, The Netherlands). All the other media and solvents used for cell culture and differentiation were purchased from Sigma-Aldrich (Schnelldorf, Germany). Organo-tin stabilized polyvinyl chloride (PVC-org. Sn) was supplied by US Pharmacopeia (Rockville, MD, USA).

### 2.2. Cell Culture

The differentiation of SH-SY5Y neuroblast-like cells into neurons was carried out following a protocol previously reported [25]. Briefly, SH-SY5Y cells at the third passage were split upon reaching approximately 70–80% confluency. A 2 mL solution of 0.05% trypsin–EDTA (1×) was added, and the cells were incubated for three minutes. Trypsin activity was neutralized by adding 10 mL of basic growth medium (BGM). The cell suspension was transferred to a 15 mL conical tube and centrifuged at 1000 rcf for 2 min at room temperature. The supernatant was carefully removed, and the pellet was resuspended in 5 mL of BGM. Cell counting was performed using a hemocytometer, and the suspension was diluted in BGM to a concentration of 50,000 cells/mL. A 2 mL aliquot of this suspension (100,000 cells) was plated into each 35 mm^2^ Petri dish and incubated. The differentiation protocol proceeded according to the schedule reported in Table 1.

### 2.3. NMR Metabolomics

#### 2.3.1. Aβ, Lycorine, and Galantamine Exposure of SH-SY5Y

The differentiated SH-SY5Y cells were exposed to Aβ 2 µM, both in the absence and the presence of the LYC and GAL at different concentrations reported in Table 2. The selected concentrations were determined after collecting data at varying concentrations. For GAL and LYC, the concentrations were chosen based on their ability to protect against Aβ-induced toxicity, as previously reported [20]. Specifically, LYC concentrations of 8 µM and 1.6 µM and GAL concentrations of 500 µM and 250 µM were selected, as they effectively mitigated the toxic effects of Aβ, with LYC showing a stronger protective effect at the lower concentrations. Stock solutions of Aβ were prepared in 60% ethanol, while those of LYC and GAL were prepared in distilled water. Each was diluted in DM3 to achieve the desired working concentrations reported in Table 2. A total of 2 mL of the test solutions was added to each Petri dish containing differentiated SH-SY5Y cells and incubated for 24 h at 37 °C with 5% CO_2_.

After incubation, cell viability was assessed using the Neutral Red uptake (NRU) assay, a method based on the uptake and lysosomal accumulation of the dye in viable cells, providing a quantitative measure of cell viability. This evaluation was included to complement the metabolomic analysis by allowing for a correlation between the metabolic alterations and cell viability rather than serving as an independent outcome measure. Each experimental condition was tested with a minimum of three replicates. Following the viability assessment, the medium was collected, and the dishes were washed with cold PBS before the solvent extraction procedure described below. Unexposed cells were used as controls.

The NMR-based metabolomics analysis was performed on the growth media of SH-SY5Y cells unexposed and exposed to the compounds reported in Table 2, with a total of six separated conditions (each consisting of a minimum of three replicates). A total of 2 mL of the test solutions was added to each Petri dish containing differentiated SH-SY5Y cells and incubated for 24 h at 37 °C with 5% CO_2_. As the control, untreated cells were grown in parallel for the same time period. At the end of the incubation time, 1 mL of the cellular medium was collected and stored at −30 °C until the registration of the NMR spectra.

#### 2.3.2. NMR Data Acquisition and Processing

Two different approaches were used for the NMR analysis of the cell medium. In the first method, 540 µL of the cell medium were directly analyzed after the addition of 60 µL of D_2_O containing 1.1 mM TMSP-d_4_ in the NMR tube. In the second approach, the cell medium was first subjected to lyophilization until complete dehydration was achieved. This step was performed to ensure sample integrity for NMR spectral acquisition, particularly considering that the samples were shipped prior to analysis. The resulting dried residue (10 mg) was then reconstituted in 600 µL of D_2_O, ensuring complete dissolution through gentle mixing. The reconstituted sample was subsequently transferred into an NMR tube for spectral acquisition. The pH of each sample was adjusted and standardized to 8.0 across all NMR tubes.

The NMR spectra of each replicate condition were acquired using both a Bruker Avance III 800 and 600 MHz spectrometers (Bruker BioSpin, Billerica, MA, USA) operating at a controlled temperature of 298 ± 0.1 K. The spectrometers were equipped with a 5 mm BBI probe featuring a *z*-axis gradient coil and an automatic tuning and matching (ATM) system. The spectra of the growth media were recorded using a 1D nuclear Overhauser enhancement spectroscopy (NOESY) presaturation pulse program or an excitation-sculpting water suppression pulse program. The acquisition parameters included 64 or 128 scans, 65,536 data points, a spectral width of 14 ppm, and a relaxation delay of 5 s. NMR data post-processing, including peak calibration and phase correction, was performed using both TopSpin 3.6.4 (Bruker, Bremen, Germany) and MestReNova v 14.3.0-30573 software (Mestrelab Research, A Coruña, Spain). Binning was carried out using a 0.04 ppm bin width with the average sum method.

#### 2.3.3. Multivariate Analysis and Spectral Integration

To comprehensively evaluate the metabolic profiles and variations associated with both the toxic and protective effects of Aβ and the two alkaloids, GAL and LYC, a combination of univariate and multivariate statistical analyses was applied to identify the metabolites involved in the protective mechanisms of the two alkaloids. In particular, a principal component analysis (PCA), a partial least squares discriminant analysis (PLS-DA), and an orthogonal partial least squares discriminant analysis (ortho-PLS-DA) were performed. PCA, an unsupervised chemometric technique, was initially applied using an untargeted approach, aiming to explore the main sources of variance in the dataset and identify global metabolic trends. In addition, PCA, PLS-DA, and ortho-PLS-DA were performed using a supervised approach based on the identification of the metabolites most involved in cellular differentiation and energy metabolism. This analysis aimed to enhance group separation by focusing on the metabolite variations specifically linked to the protective effects of GAL and LYC. To ensure quantitative accuracy, the integral areas were normalized relative to the concentration of TMSP present in the solution. Subsequently, statistical analyses were performed using both the MetaboAnalyst 6.0 [26] and the Python script scikit-learn, a library designed for machine learning and statistical analysis [27]. The integration of Python 3.12 libraries was adopted to ensure enhanced numerical stability, considering the high number of ppm values involved, which increases the risk of rounding errors and computational instability. Prior to analysis, the data were normalized using Pareto scaling, ensuring comparability across different experimental conditions.

## 3. Results

**Figure 2:**
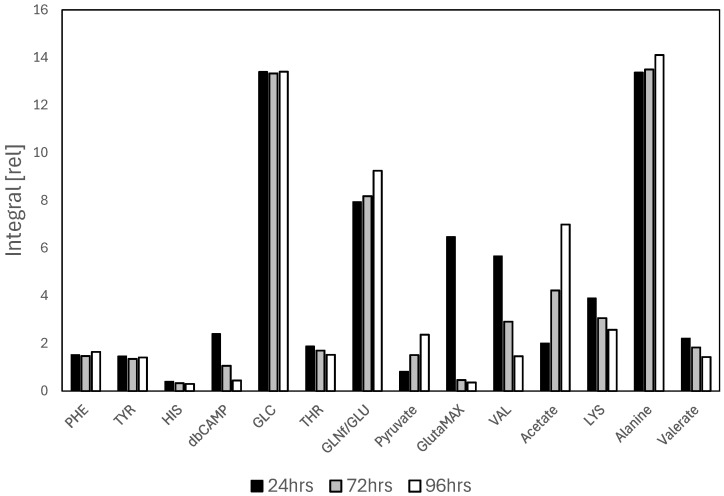
Relative integrals of selected metabolites measured by NMR in the culture medium of SH-SY5Y cells incubated for 24, 72, and 96 h. The integral [rel] values are normalized relative to the NMR signal area of TMSP-d_4_, used as an internal reference standard.

The extracellular metabolism of non-differentiated SH-SY5Y cells was previously investigated by analyzing changes in metabolite levels in the cellular medium after incubation with the cells. Both high-resolution liquid-state and magic-angle spinning (MAS) NMR studies revealed selective patterns of metabolite consumption and excretion [28,29]. The metabolites observed in differentiated SH-SY5Y cellular medium, in this study, were very similar to those of non-differentiated cells, with the exception of the metabolites present in the culture medium used for the differentiation process. In particular, the most significant variations were observed for the metabolites db-cAMP and GlutaMAX, whose characteristic signals are detected in the NMR spectrum (Figure S1). Specifically, it was observed that the signals of these metabolites are progressively consumed as the incubation time of the cells increases, accompanied by simultaneous changes in other metabolites closely linked to energy consumption and neuronal activity. The key metabolites highlighted in Figure 2 include db-cAMP, GlutaMAX, glutamine (GLN), glutamate (GLU), pyruvate, acetate, lactate, lysine (LYS), and threonine (THR). The signals corresponding to db-cAMP are prominently detected across various regions of the spectrum, reflecting its role as a cyclic AMP analog associated with differentiation processes (Figure S1). Its consumption is closely linked to the ongoing differentiation occurring during the incubation period. Simultaneously, the consumption of GlutaMAX and the excretion of GLU reflect the glutamine–glutamate dynamics that are critical for neuronal function and energy metabolism. Pyruvate, as an intermediate in the tricarboxylic acid (TCA) cycle, further underscores their relevance to energy production. While the differentiation of SH-SY5Y cells increases their relevance for specific studies by better replicating neuronal characteristics, it is a time-consuming and resource-intensive process. The complexity of the differentiation protocols, coupled with the prolonged culture periods required to achieve a stable phenotype, can ultimately restrict the number of samples that can be processed and analyzed, posing challenges to the scalability of experiments.

The ^1^H NMR spectra of the cellular medium from the SH-SY5Y cells exposed to different treatments were recorded to evaluate the specific metabolic changes induced by GAL and LYC, which have previously been shown to exert neuroprotective effects on Aβ toxicity [20]. The NMR spectra recorded for the samples with GAL and LYC-containing treatments did not show any signals belonging to LYC and GAL protons (even in spectra recorded with NS = 1024), strongly suggesting intracellular uptake of both GAL and LYC.

To comprehensively evaluate the metabolic effects associated with the protective role of GAL and LYC against Aβ toxicity, a detailed analysis was performed by combining univariate and multivariate statistical approaches.

PCA was first performed using an untargeted approach on the full NMR spectra (excluding the water regions) of samples from the control group, Aβ-exposed cells, LYC + Aβ-exposed cells, and GAL + Aβ-exposed cells. The initial analysis did not show clear classification due to high data variability. The analysis was then refined to focus on Aβ-exposed samples and those treated with LYC or GAL only, analyzing the LYC and GAL groups separately to better capture their distinct metabolic effects. At 24 h, PCA revealed distinct metabolic profiles between the groups (Figure S2). In the LYC-treated samples, Aβ formed a separate cluster from the LYC-treated samples, suggesting that Aβ alone induces a unique metabolic profile. Both LYC concentrations were positioned apart from the Aβ, suggesting LYC modulation of the metabolic profile, with different concentrations inducing distinct metabolic changes. This clustering suggests that Aβ alone creates a unique metabolic disturbance, which lycorine modulates, potentially reflecting its neuroprotective or anti-aggregation properties. Similarly, PCA analysis of the cellular media exposed to Aβ and treated with GAL revealed that Aβ formed a distinct cluster from GAL-treated samples, further supporting a unique metabolic profile associated with Aβ toxicity. The two GAL concentrations were clearly separated from the Aβ cluster, with GAL 0.5 mM showing a more pronounced shift along both PC1 and PC2. The findings from the PCA analysis align closely with the cell viability results previously reported [20]. Specifically, the data for the samples analyzed by PCA, presented in Table 3, indicate that LYC at 1.6 µM and GAL at 500 µM represent the highest effective concentrations—relative to each alkaloid—capable of restoring SH-SY5Y cell viability after Aβ exposure.

In order to identify the metabolites involved in the neuroprotective function of the two alkaloids, we restricted the statistical analysis to focus on the metabolites associated with the normal activity of differentiated SH-SY5Y cells, as described and reported in Figure 2. For this analysis, NMR spectra corresponding to the control group, Aβ-exposed cells, LYC + Aβ-exposed cells, and GAL + Aβ-exposed cells were all considered. The selected metabolites were further analyzed using an ANOVA, which demonstrated strong statistical significance, with *p*-values lower than 0.01 for most of them.

**Figure 3:**
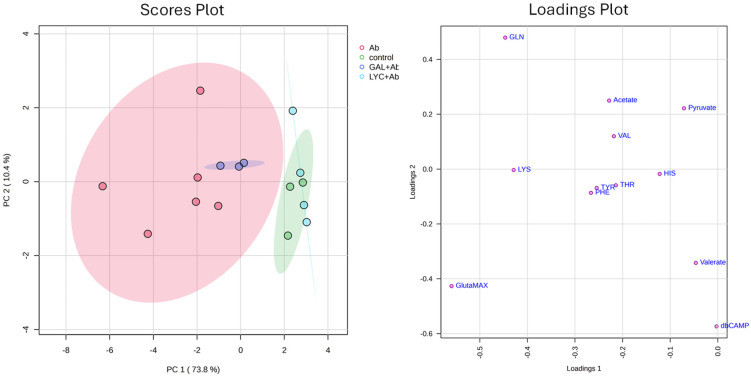
Principal component analysis (PCA) plots illustrating the effects of exposure of Aβ42 alone or in presence of LYC and GAL on the metabolic profiles of SH-SY5Y cells. The left panel (Scores Plot) illustrates the distribution of different experimental groups based on PCA analysis. Each point represents a sample, with different colors corresponding to the following conditions: Aβ42 (red), control (green), GAL + Aβ42 (blue), and LYC + Aβ (light blue). The ellipses indicate 95% confidence regions for each group. PC1 and PC2 explain 73.8% and 10.4% of the variance, respectively. The right panel (loadings plot) displays the contribution of specific metabolites to the PCA model, where metabolites such as GLN, GlutaMAX, and LYS are the primary contributors to the variance observed in the scores plot.

**Figure 4:**
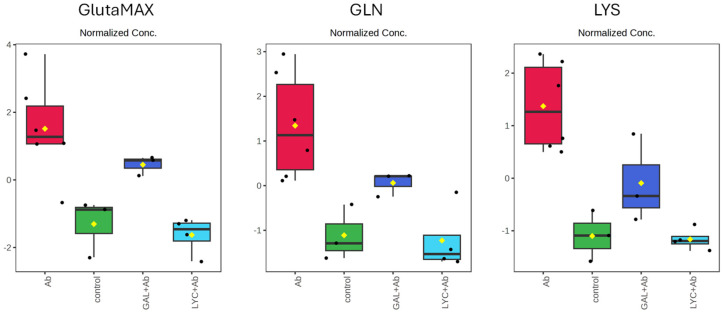
Box plots of normalized metabolite concentrations across experimental conditions. Each panel represents the distribution of a specific metabolite among the four experimental groups: Aβ42-treated (red), control (green), GAL + Aβ42 (blue), and LYC + Aβ42 (light blue). The yellow diamonds indicate the mean values, while black dots represent individual data points.

PCA was initially performed, and the results, represented by the scores plot and loadings plot, are shown in Figure 3. The scores plot reveals an R^2^ of 0.73 and a *p*-value < 0.05, indicating that the model explains a significant portion of the variance in the data and achieves statistical significance. The distribution of individual samples along the first two principal components (PC1 and PC2) together explains 84.2% of the total variance, respectively. The Aβ42 group exhibits the greatest dispersion, reflecting elevated metabolic variability, whereas the control, GAL + Aβ42, and LYC + Aβ42 groups display more compact distributions. The Aβ42-treated group (red) forms a distinct cluster with broad dispersion along PC1, indicating substantial metabolic fluctuations in response to Aβ42 exposure. In contrast, the control (green) and LYC + Aβ42 (light blue) groups cluster separately and remain distant from the Aβ42-treated group, suggesting a pronounced metabolic shift induced by the treatment. The loadings plot (Figure 3, right panel) identifies the specific metabolites contributing to the separation observed in the scores plot. Each point represents a metabolite, and its position along PC1 and PC2 indicates its influence on sample clustering. GLN, GlutaMAX, and LYS are positioned significantly along Loadings 1, suggesting a major influence on group differentiation, particularly along PC1. In particular, their positioning suggests that they are key drivers of the metabolic differences observed among the experimental groups. The variations in their concentrations across the four sample types are displayed in Figure 4. Notably, for all three metabolites, exposure of the cells to Aβ42 leads to an increase in their concentration in the culture medium compared to the negative control values. In contrast, GAL and LYC reverse this behavior by decreasing the metabolite concentrations. Specifically, in the case of LYC, the metabolite levels return to approximately those observed in the negative control, which is in agreement with the cellular viability reported in Table 3.

**Figure 5:**
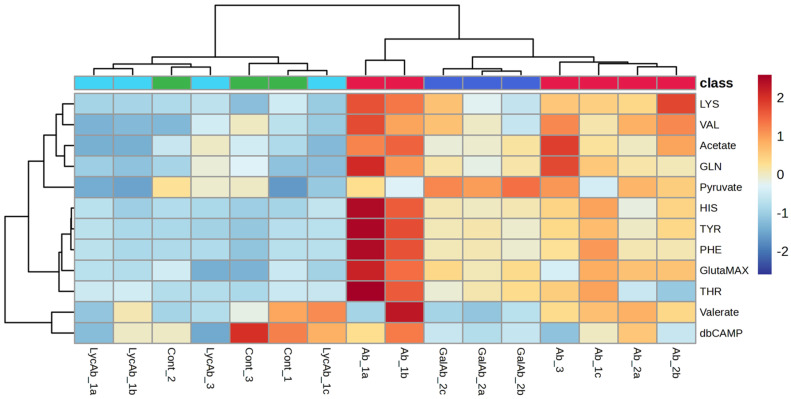
Heatmap with hierarchical clustering showing metabolite concentration variations across different sample groups: Aβ42-treated (red), control (green), GAL + Aβ42 (blue), and LYC + Aβ42 (light blue). Rows represent metabolites, while columns correspond to individual samples (grouped by experimental conditions. The color scale indicates relative metabolite abundance, with red representing higher concentrations, blue indicating lower concentrations, and neutral colors reflecting intermediate levels.

The same metabolites were also identified as key VIP (variable importance in projection) scores (VIP > 1) in the PLS-DA analysis performed on the same dataset (Figure S3). This further supports their relevance in distinguishing different experimental conditions, reinforcing their role as metabolic markers in response to the Aβ42, GAL + Aβ42, and LYC + Aβ42 treatments. In particular, the heatmap with hierarchical clustering reported in Figure 5 provides a visual representation of metabolite variations across different experimental conditions, offering a clear overview of how metabolites change in response to treatments. The columns correspond to different samples, which appear to be grouped by experimental conditions, while the rows represent specific metabolites. The color scale indicates the relative concentration of each metabolite, where red shades represent higher concentrations, blue shades indicate lower concentrations, and neutral colors (yellow) reflect intermediate levels. The dendrograms on both axes illustrate hierarchical clustering, grouping samples and metabolites with similar profiles together. In particular, Figure 5 shows the presence of two distinct sample clusters: one for the control and LYC + Aβ42 samples and the other for the Aβ42 and GAL + Aβ42 samples. The first cluster, which includes the control and LYC + Aβ42 samples, is predominantly characterized by blue-colored metabolites, reflecting lower metabolite concentrations. Conversely, the second cluster, consisting of Aβ42 and GAL + Aβ42 samples, is mainly represented by red-colored metabolites, indicating higher metabolite levels. However, exceptions to this general trend are observed for acetate, THR, and pyruvate, exhibiting larger variability across all the samples.

**Figure 6:**
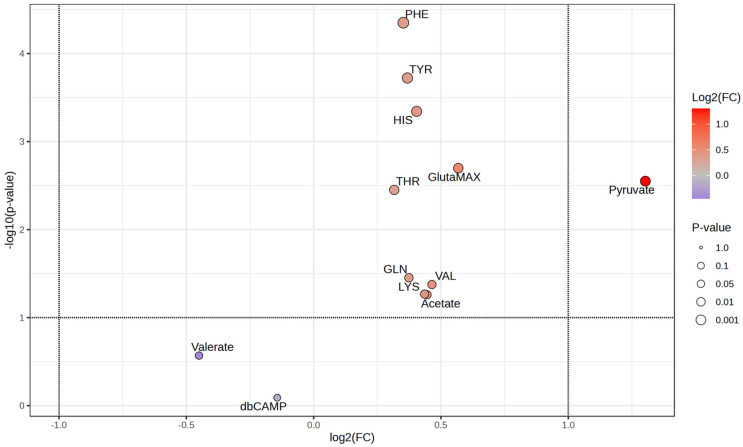
Volcano plot representing metabolite differences between experimental groups. The *x*-axis shows the log2 fold change (log2(FC)) of metabolite abundance, while the *y*-axis represents the log10 (*p*-value), indicating statistical significance. Metabolites with higher significance appear toward the top of the plot. Color intensity corresponds to log2(FC), with red indicating upregulated metabolites and purple indicating downregulated ones. The dot size reflects the *p*-value, where larger points indicate higher significance. Pyruvate exhibits the most significant upregulation, while Valerate and db-cAMP show a trend towards downregulation with very low statistical significance. The dashed horizontal line represents the threshold for statistical significance.

Considering the differences observed between the samples treated with GAL and LYC, we subsequently subjected the data corresponding to these samples to both univariate and multivariate statistical analyses. The goal was to identify metabolites associated with these two molecules that could help explain the observed and different cellular viability behavior. Initial analysis using the volcano plot (a graphical tool that visualizes statistical significance and magnitude of fold changes between groups) revealed that pyruvate was the most significant metabolite, both in terms of *p*-value and fold change (Figure 6).

**Figure 7:**
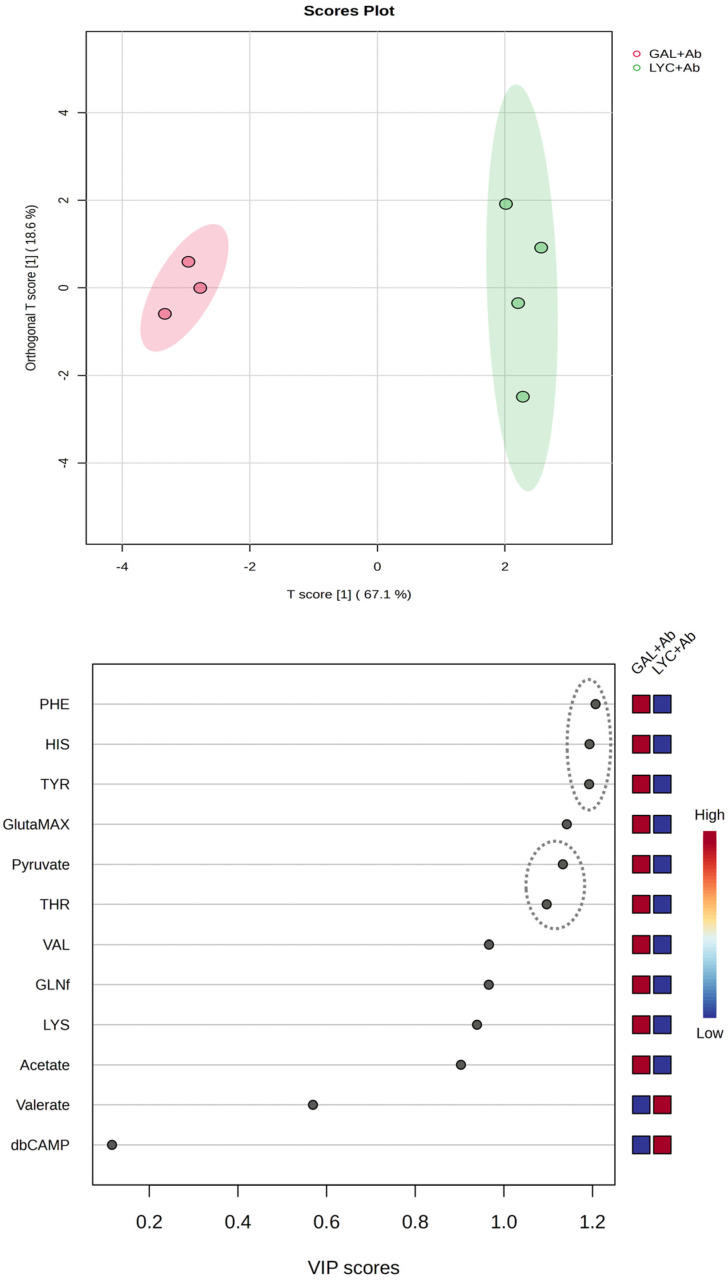
Orthogonal partial least squares discriminant analysis (O-PLS-DA) model evaluating the variance between GAL + Aβ and LYC + Aβ groups (R^2^X = 0.671, R^2^Y = 0.964, Q^2^ = 0.927). The scores plot (top) shows a clear separation between the two groups along the first component (T score [1], 67.1%; R2), with an orthogonal component (orthogonal T score [1], 18.6%) capturing non-predictive variance. The VIP scores plot (bottom) highlights the most relevant metabolites contributing to class differentiation. Newly identified metabolites with VIP scores > 1 are indicated with circles.

As expected, the PLS-DA analysis performed on these two samples led to conclusions very similar to those obtained for the full dataset containing control and Aβ42 samples (Figure S4). Among the VIP scores, the top three metabolites remain relatively unchanged. However, one of the most significant variations observed is the increased significance of pyruvate. This metabolite exhibits the most pronounced change, shifting from a VIP score close to zero to approximately 1. The importance of pyruvate also emerged from the Ortho-PLS-DA analysis, identifying PHE, HIS, TYR, pyruvate, and TYR as additional VIP-scores explaining the variance between the LYC + Aβ42 and GAL + Aβ42 samples (Figure 7). Such differences might be explained by considering that Ortho-PLS-DA, although being an extension of PL-SDA, incorporates an additional filtering step to remove orthogonal variance by improving the interpretability of the model by enhancing class separation and reducing noise from the confounding variables [30]. The Ortho-PLS-DA model used for classification exhibits strong explanatory and predictive performance, with cross-validation parameters indicating a well-fitted model (R^2^X = 0.671, R^2^Y = 0.964, and Q^2^ = 0.927). The high Q^2^ value suggests a robust predictive capability, supporting the distinct clustering observed in the scores plot.

## 4. Discussion

The intricate interplay of pathological events characterizing the multifactorial nature of AD poses significant challenges to the development of effective treatments, making model systems indispensable tools for identifying individual mechanisms and their interactions [31]. At the same time, model systems serve as crucial platforms for evaluating the efficacy of potential therapeutic and diagnostic interventions in a controlled and reproducible manner [32,33,34,35]. The SH-SY5Y cell line, derived from human neuroblastoma, is widely used as an in vitro model for studying AD. Its relevance lies in its ability to differentiate into neuron-like cells under specific conditions, mimicking key neuronal characteristics and enabling the investigation of the molecular and cellular mechanisms involved in neurodegeneration [36,37,38,39]. Differentiated SH-SY5Y cells exhibit functional properties of mature neurons, including synaptic protein expression, neurotransmitter synthesis, and active mitochondrial metabolism [40,41]. Moreover, SH-SY5Y cells provide a suitable platform for modeling the accumulation of Aβ aggregation, elevated levels of reactive oxygen species (ROS), and impaired metal ion homeostasis, known as typical AD features. In fact, SH-SY5Y cells are sensitive to oxidative stress, have the ability to respond to amyloidogenic peptides, and reflect neurotoxic changes in vitro [42]. A major advantage of SH-SY5Y cells is their adaptability to various experimental designs. For instance, their differentiation using agents like retinoic acid (RA) or brain-derived neurotrophic factor (BDNF) enhances their neuronal phenotype, making them more representative of human neurons [43]. Differentiated cells exhibit increased mitochondrial activity, axonal growth, and neurotransmitter release, enabling more precise studies of AD-related processes such as tau phosphorylation, Aβ aggregation, and synaptic dysfunction [40].

As shown in Figure 2, db-cAMP, used for cell differentiation to promote neurite outgrowth, is normally taken up by SH-SY5Y cells and hydrolyzed by cellular esterases to release active cAMP, a key secondary messenger involved in numerous cellular processes [44]. In particular, the NMR signals of db-cAMP are consistently decreased over time, indicating the importance of this molecule for cellular activity. In addition to db-cAMP, GlutaMAX is also extensively consumed during the normal SH-SY5Y activity (Figure 2). GlutaMAX^TM^ is a stable dipeptide form of L-glutamine (L-alanyl-glutamate) and is commonly used in cell culture media for SH-SY5Y differentiation. Glutamine is an essential amino acid that is required for various cellular processes, including protein synthesis, cell division, and energy metabolism. However, in culture media, L-glutamine is prone to degradation, especially in long-term cultures, which can lead to reduced cell growth and viability. To overcome this limitation, GlutaMAX is added to SH-SY5Y culture media as a more stable alternative to L-glutamine. GlutaMAX provides the same metabolic benefits as L-glutamine but remains stable in solution for longer periods, preventing the degradation issues associated with free glutamine. This is crucial for maintaining the health and functionality of SH-SY5Y cells during extended culture periods.

The metabolic profile of differentiated SH-SY5Y cells also reveals unchanged glucose level (Figure 2), along with a concomitant increase in pyruvate and acetate, suggesting a metabolic shift likely associated with a quiescent or differentiated state. The stable glucose concentration indicates that its uptake and utilization remain balanced, possibly due to an adaptation of metabolic fluxes rather than a change in overall glucose consumption. The accumulation of pyruvate might result from a reduction in its conversion to lactate or a bottleneck in mitochondrial oxidative metabolism, potentially limiting its entry into the tricarboxylic acid (TCA) cycle. The increased acetate levels could be attributed to enhanced lipid catabolism or alterations in acetyl-CoA metabolism, possibly linked to a more oxidative state. Acetate is a known byproduct of lipid oxidation, amino acid degradation, and acetyl-CoA hydrolysis, and its accumulation suggests that lipid utilization might be contributing significantly to the cellular energy balance. These observations align with the quiescent phenotype induced by B-27 supplementation, which is known to support neuronal differentiation while reducing cell proliferation. Cells in a quiescent state typically exhibit lower energy demands, a decrease in glycolytic flux, and a greater reliance on oxidative metabolism [45].

To evaluate the effects of Aβ42 exposure both in the presence and in the absence of the GAL and LYC alkaloids, a statistical analysis on the extracellular metabolite profiles extracted from the NMR spectra of the cellular medium was performed. The untargeted metabolomic analysis enabled an evaluation of the effects of LYC and GAL on the full metabolite profiles (Figure S2). Both LYC and GAL exhibited distinct separation from the Aβ-treated condition, demonstrating their ability to mitigate the metabolic shifts induced by Aβ exposure and their shared influence on the metabolic response to Aβ. In both cases, increasing concentrations of the compounds (1.6 µM to 8 µM for LYC, and 250 µM to 500 µM for GAL) resulted in distinct positions on the PCA plots, indicating dose-dependent modulation of the cellular metabolic profile. The obtained findings point out the ability of both alkaloids to effectively counteract the metabolic dysregulation induced by Aβ, being that their effects are distinct in both magnitude and mechanism. These differences likely arise from variations in their mechanisms of action, including how they influence biochemical pathways and interact with the cellular metabolic network to regulate key processes.

To better evaluate the metabolic pathways associated with GAL and LYC activities, a statistical analysis on the most significant metabolites was carried out. Our data indicate that both GLN and GlutaMAX levels are strongly altered in differentiated SH-SY5Y cells upon Aβ exposure (Figure 3 and Figure 4). Glutamine is a critical amino acid involved in multiple biological processes, including energy metabolism, neurotransmission, and cellular redox balance. It serves as a precursor for glutathione, the most abundant antioxidant in cells, and plays a key role in the glutamine–glutamate cycle, which is crucial for neuronal function. Additionally, glutamine acts as an alternative carbon source for the TCA cycle, and its altered metabolism is associated with mitochondrial dysfunction [46]. As the most abundant amino acid in the brain, glutamine is essential for synaptic transmission, plasticity, and energy metabolism [47]. It also contributes to neurotransmitter synthesis, neuronal signaling regulation, and intracellular osmotic balance [48]. Furthermore, its role in glutamate homeostasis is critical for cognitive functions such as learning and memory, which depend on finely tuned excitatory neurotransmission [49].

In SH-SY5Y cells, glutamine is indispensable for maintaining cellular viability and supporting the differentiated neuron-like phenotype [50]. The role of GLN as a key nutrient for SH-SY5Y cell growth has been previously documented in the literature, as has the significant reduction in cell viability upon Aβ exposure [51]. Similarly, our data indicate a reduced consumption of GLN in the Aβ42 samples, suggesting a correlation between the impaired metabolism of this essential amino acid and Aβ-induced toxicity. This is also supported by the fact that the co-administration of LYC and Aβ42 restores GLN and GlutaMAX levels to the basal values observed in the negative control, thereby justifying the observed protective role of LYC on cell viability (Figure 4). A similar effect is observed with GAL, although it exhibits a less-pronounced impact compared to LYC. Consistent with these findings, the previously measured cell viability values for the LYC–Aβ system are larger than those for the GAL–Aβ system (Table 3).

Aberrant glutamine metabolism is also increasingly recognized as a key factor in AD, impacting glutamate neurotransmission [52]. Studies have reported disruptions in glutamine–glutamate homeostasis, with reduced cortical glutamate levels and increased glutamine in AD patients, suggesting an impaired glutamine–glutamate cycle [53]. In vivo, MRSs further confirm decreased cortical glutamate concentrations in AD patients [54].

In addition to glutamine, our data highlight lysine as another key metabolite associated with Aβ-induced toxicity in SH-SY5Y cells. Interestingly, recent metabolomic studies have also identified lysine metabolism as a crucial pathway altered in AD, implicating its role in oxidative stress, mitochondrial dysfunction, and epigenetic regulation. In particular, a comparative brain metabolomic analysis has associated mitochondrial dysfunction with lysine metabolism [55]. Further evidence from MS-based metabolomics of postmortem superior frontal gyrus tissue revealed significant disturbances in lysine degradation [56], reinforcing its role in acetyl-CoA production, energy metabolism, and neurotransmitter synthesis. Finally, a study on serum and cerebrospinal fluid (CSF) metabolism demonstrated that lysine metabolism was significantly disrupted in individuals with mild cognitive impairment (MCI), indicating its potential as an early biomarker for AD progression [57]. Collectively, these findings suggest that lysine metabolism is intricately linked to mitochondrial health, oxidative stress, and epigenetic regulation.

The two alkaloids also revealed a significant statistical difference in pyruvate levels in the cellular media, with higher levels observed in the GAL + Aβ samples (Figure 6). Pyruvate plays a crucial role in energy production, oxidative stress management, and neuroprotection. Several studies have demonstrated that pyruvate metabolism is significantly dysregulated in AD, contributing to neuronal dysfunction and disease progression [58]. Pyruvate serves as a critical substrate for mitochondrial energy metabolism, as it is converted into acetyl-CoA by the pyruvate–dehydrogenase complex (PDHC) to fuel the TCA cycle. However, studies have shown a significant decrease in PDHC activity in AD brains, along with reduced activity of other key mitochondrial enzymes, such as α-ketoglutarate dehydrogenase (α-KGDHC) [58,59]. The impairment of mitochondrial metabolism is also exacerbated by Aβ accumulation, which has been shown to disrupt synaptic plasticity and neuronal energy homeostasis [60].

Beyond its role in energy metabolism, pyruvate has been recognized for its neuroprotective properties as well. Previous studies on SH-SY5Y neuroblastoma cells demonstrated that pyruvate protects CNS neurons from oxidative stress [61,62], reduces inflammation [63], enhances erythropoietin (EPO) expression contributing to neuronal survival and repair [64], and protects cells from glutamate excitotoxicity [65]. The interaction between pyruvate metabolism and autophagy is further supported by another study demonstrating pyruvate-induced activation of mitophagy, which restores mitochondrial function and protects SH-SY5Y cells from apoptotic and necrotic death induced by Aβ toxicity [66].

Untargeted metabolomics on hippocampal samples also revealed that pyruvate metabolism, glycolysis/gluconeogenesis, and amino acid metabolism (Ala/Asp/Glu pathways) were among the most significantly downregulated pathways in AD [67]. Additionally, pathway topology analysis revealed that pyruvate metabolism and the TCA cycle were altered in multiple brain regions, particularly in the prefrontal cortex, hippocampus, and superior temporal gyrus [68]. Finally, pyruvate has been identified as a potential biomarker for AD. Elevated levels have been reported in CSF [69], and more recently, serum metabolic signatures in AD patients have shown alterations in pyruvate-associated pathways [70].

Monitoring pyruvate levels and flux in SH-SY5Y cells treated with GAL and LYC reveals distinct metabolic impacts. While LYC treatment maintains pyruvate concentrations close to the basal control values, even in the presence of Aβ, GAL treatment leads to a marked accumulation of pyruvate. This differential pattern indicates that LYC may better preserve mitochondrial function and energy metabolism under Aβ-induced stress, offering valuable insights for developing targeted therapeutic strategies.

## 5. Conclusions

This study provides valuable insights into metabolic dysregulation in AD and the potential therapeutic effects of GAL and LYC in counteracting these alterations. Using SH-SY5Y cells, a well-established in vitro neurodegeneration model, we evaluated the metabolic response to Aβ toxicity in the presence and absence of the two alkaloids. The results reveal distinct metabolic effects of GAL and LYC, particularly in their ability to restore glutamine homeostasis, modulate pyruvate metabolism, and support mitochondrial function. Exposure to Aβ42 significantly disrupts glutamine metabolism, reinforcing its key role in neuronal energy balance and neurotransmission. Our findings indicate that both LYC and GAL restore glutamine levels, with LYC demonstrating a more pronounced effect. In addition to glutamine, our data highlight lysine as another key metabolite associated with Aβ-induced toxicity in SH-SY5Y cells. The ability of LYC and GAL to influence lysine-associated pathways suggests a broader metabolic impact, potentially contributing to the regulation of mitochondrial health and neuronal survival. Finally, the comparison between the two alkaloids highlights their differential impact on pyruvate metabolism, a critical pathway impaired in AD. LYC is particularly effective in maintaining pyruvate and glutamine homeostasis, suggesting a greater influence on mitochondrial function and oxidative metabolism. In contrast, GAL treatment leads to pyruvate accumulation, possibly indicating a shift toward glycolysis or a cellular protective response against oxidative stress. These differences suggest distinct mechanisms of action, which may be critical for determining their therapeutic potential and effectiveness in mitigating AD-related neurodegeneration. Further studies should focus on elucidating the precise molecular mechanisms underlying these metabolic effects, with a particular emphasis on mitochondrial function, oxidative stress regulation, and metabolic adaptation in neuronal models of AD.

In light of the increasing interest in therapeutic strategies for AD, LYC’s potential stands out due to its broad spectrum of biological activities, particularly its ability to target the various factors involved in the disease’s complex etiology. The possible synergy between LYC and GAL (or other compounds) offers an exciting direction for future research. Such an approach could not only enhance the shared neuroprotective effects but also leverage the unique properties of each compound, potentially leading to more effective treatments for AD.

## Figures and Tables

**Table 1:** Protocol applied for SH-SY5Y differentiation. The differentiation of SH-SY5Y cells was carried out following a standardized protocol to ensure neuronal-like characteristics suitable for experimental analysis [25].

Day	Procedure
Day 1	Replace BGM with Differentiation Medium 1 (DM1) *.
Day 3	Change DM1 with new medium.
Day 6	Change DM1 with new medium.
Day 7	Split cells, suspend in DM1, and replate into new 35 mm^2^ Petri dishes.
Day 8	Replace DM1 with Differentiation Medium 2 (DM2) **.
Day 10	Split cells, suspend in DM2, and seed into extracellular matrix (ECM)-coated 35 mm^2^ plates.
Day 13	Replace DM2 with Differentiation Medium 3 (DM3) ***.
Day 14	Change DM3 with new medium.
Day 17	Change DM3 with new medium.
Day 18	Neuronal cultures are ready for experiments.

* DM1 composition is Eagle’s Minimum Essential Medium (EMEM), 2.5% hiFBS, 1× Pen/Strep, 2 mM Glutamine, 10 μM RA. ** DM2 composition is EMEM, 1% hiFBS, 1× Pen/Strep, 2 mM Glutamine, 10 μM RA. *** DM3 composition is neurobasal, 1× B27, 20 mM KCl, 1× Pen/Strep, 2 mM Glutamax, 50 ng/mL BDNF, 2 mM dibutyryl cyclic AMP (db-cAMP), 10 μM RA.

**Table 2:** Compounds and concentrations exposed to differentiated SH-SY5Y cells. GAL and LYC were co-administered in combination with Aβ.

Compound	Concentrations
Aβ	2 µM
LYC	8 µM
	1.6 µM
GAL	500 µM
	250 µM

**Table 3:** Effect of LYC and GAL on the viability of differentiated SH-SY5Y cells exposed to Aβ42. The percentage of viable cells was measured after treatment with Aβ42 (2 µM) alone or in combination with LYC (8 µM, 1.6 µM) or GAL (500 µM, 250 µM) [20].

Sample	% of Viable Differentiated SH-SY5Y Cells
LYCORINE	
Aβ42 2 µM	54 ± 2
Aβ42 2 µM + LYC 8 µM	94 ± 2
Aβ42 2 µM + LYC 1.6 µM	98 ± 3
GALANTAMINE	
Aβ42 2 µM	56 ± 2
Aβ42 2 µM + GAL 500 µM	77 ± 2
Aβ42 2 µM + GAL 250 µM	51 ± 2

## Data Availability

Data are contained within the article and the Supplementary Materials.

## Supplementary Materials

The following supporting information can be downloaded at https://www.mdpi.com/article/10.3390/cells14070525/s1: Figure S1: Selected regions of 1H NMR spectrum of the cellular media from differentiated SH-SY5Y cells incubated for 24 hours (black), 72 hours (red), and 96 hours (blue); Figure S2: Principal Component Analysis (PCA) plots illustrating the effects of LYC (A) and GAL (B) on the metabolic profiles of SH-SY5Y cells exposed to Aβ. Each point represents a distinct treatment condition: Aβ 2 µM alone (blue), Aβ + LYC at 1.6 µM (purple) or 8 µM (orange), and Aβ + GAL at 250 µM (orange) or 500 µM (purple); Figure S3: Partial Least Squares Discriminant Analysis (PLS-DA). The scores plot (left) shows the distribution of different experimental groups: Aβ42 (red), control (green), GAL + Aβ42 (blue), and LYC + Aβ (light blue). The VIP scores plot (bottom) highlights the most relevant metabolites contributing to class differentiation; Figure S4: Partial Least Squares Discriminant Analysis (PLS-DA). The scores plot (left) shows the distribution of different experimental groups: GAL + Aβ42 (red) and LYC + Aβ (green). The VIP scores plot (bottom) highlights the most relevant metabolites contributing to class differentiation.

## Abbreviations

The following abbreviations are used in this manuscript:

AD	Alzheimer’s Disease
GAL	Galantamine
LYC	Lycorine
Aβ	Amyloid-β
AChE	Acetylcholinesterase
ROS	Reactive Oxygen Species
FDA	Food and Drug Administration
EMA	European Medicines Agency
BGM	Basic Growth Medium
DM	Differentiation Medium
ECM	Extracellular Matrix
EMEM	Eagle’s Minimum Essential Medium
BDNF	Brain-Derived Neurotrophic Factor
db-cAMP	Dibutyryl cyclic AMP
RA	Retinoic Acid
TMSP-d4	3-(Trimethylsilyl)-2,2,3,3-tetradeuteropropionic Acid Sodium Salt
NOESY	Nuclear Overhauser Enhancement Spectroscopy
PCA	Principal Component Analysis
PLS-DA	Partial Least Squares Discriminant Analysis
Ortho-PLS-DA	Orthogonal Partial Least Squares Discriminant Analysis
ANOVA	Analysis of Variance
PC	Principal Component
TCA	Tricarboxylic Acid
CSF	Cerebrospinal Fluid
MIC	Mild Cognitive Impairment
PDHC	Pyruvate-Dehydrogenase Complex
α-KGDHC	α-ketoglutarate Dehydrogenase
CNS	Central Nervous System
EPO	Erythropoietin
