# Supplementary material for: NMR Metabolomic Profiling of Differentiated SH-SY5Y Neuronal Cells: Amyloid-β Toxicity and Protective Effects of Galantamine and Lycorine
Source: Cells. 2025 Apr 1;14(7):525. doi: 10.3390/cells14070525 (PMC11988681; doi:10.3390/cells14070525)
Supplement: Supplementary file 1 [file cells-14-00525-s001.zip › cells-3529328-supplementary.pdf]

SUPPLEMENTARY MATERIAL

NMR Metabolomic Profiling of Differentiated SH-SY5Y Neuronal Cells: Amyloid-β Toxicity and Protective Effects of Galantamine and Lycorine

Arian Kola [1,†], Filippo Costanti [2,†], Jordan Kahfi [3], Abdul-Hamid Emwas [4], Mariusz Jaremko [3] and Daniela Valensin [1,5,*]

1 Department of Biotechnology, Chemistry and Pharmacy, University of Siena, Via Aldo Moro 2, 53100 Siena, Italy; arian.kola@unisi.it

2 Department of Information Engineering and Mathematics, University of Siena, 53100 Siena, Italy; costanti@student.unisi.it

3 Division of Biological and Environmental Sciences and Engineering (BESE), King Abdullah University of Science and Technology (KAUST), Thuwal 23955-6900, Saudi Arabia; jordan.kahfi@kaust.edu.sa (J.K.); mariusz.jaremko@kaust.edu.sa (M.J.)

4 KAUST Core Laboratories, King Abdullah University of Science and Technology, Thuwal 23955-6900, Saudi Arabia; abdelhamid.emwas@kaust.edu.sa

5 Consorzio Interuniversitario Risonanze Magnetiche di Metalloproteine (CIRMMP), Via L. Sacconi 6, 50019 Firenze, Italy

* Correspondence: daniela.valensin@unisi.it
† These authors contributed equally to this work.

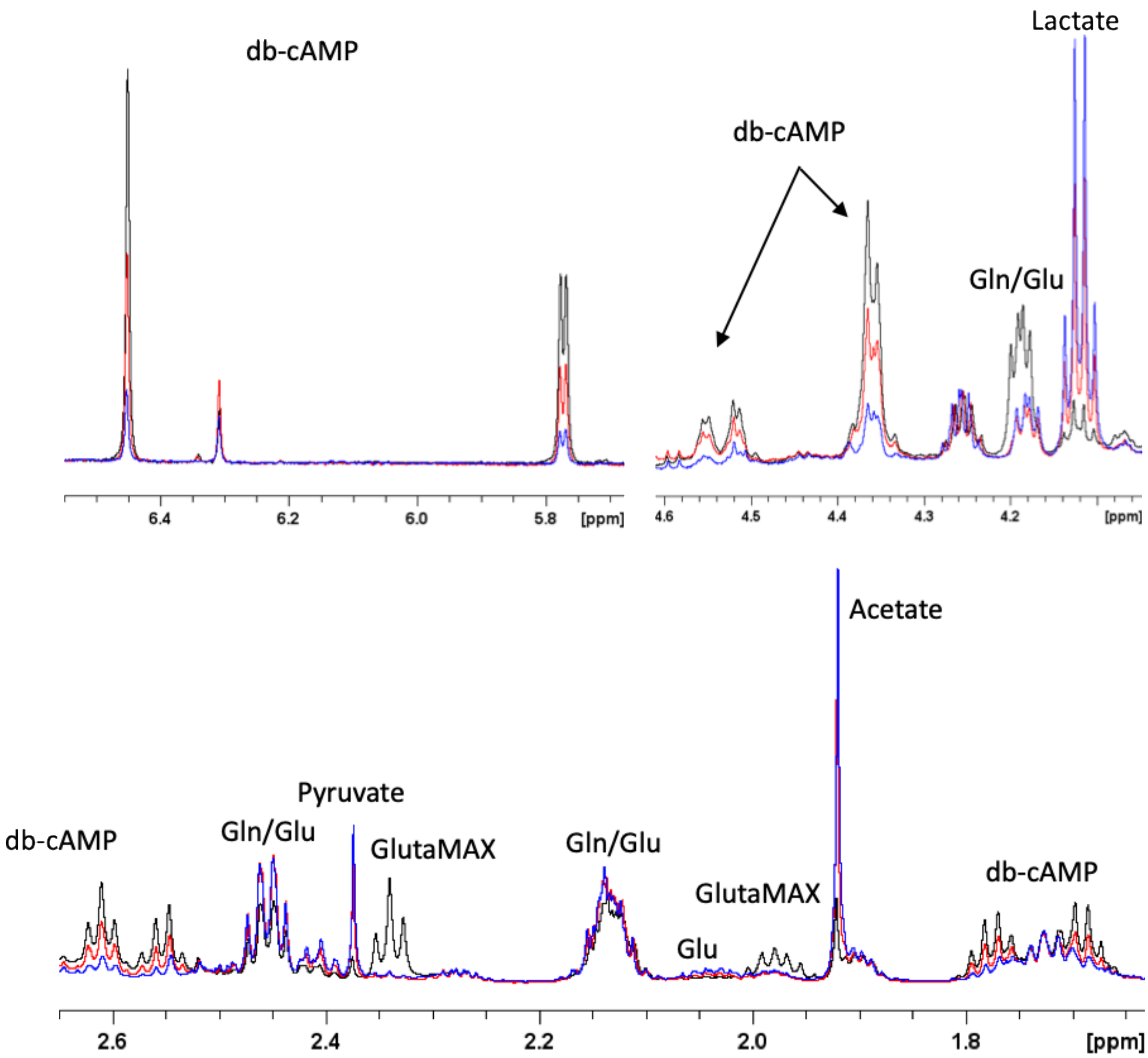

Figure S1. Selected regions of ^{1}H NMR spectrum of the cellular media from differentiated SH-SY5Y cells incubated for 24 hours (black), 72 hours (red), and 96 hours (blue).

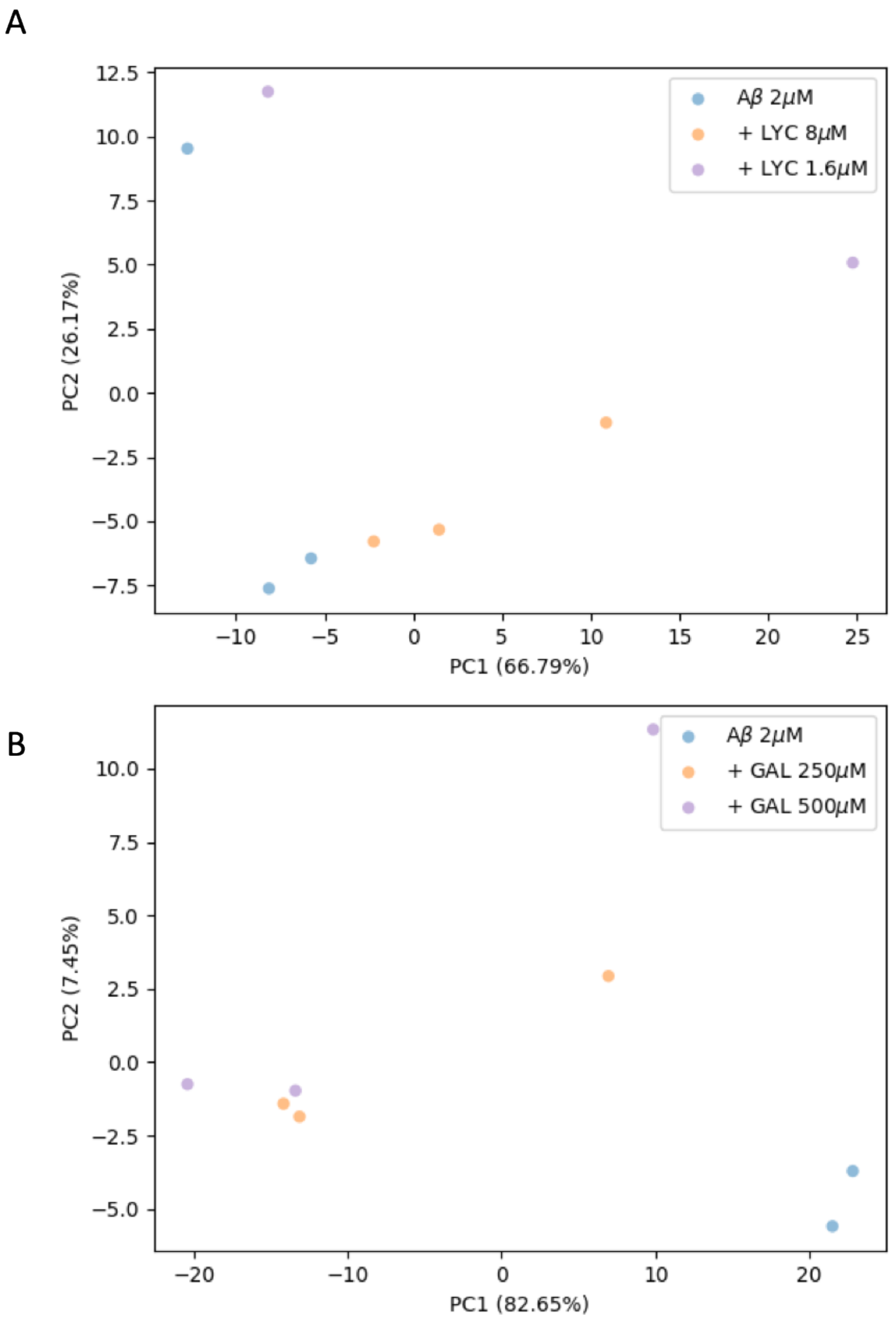

Figure S2. Principal Component Analysis (PCA) plots illustrating the effects of LYC (A) and GAL (B) on the metabolic profiles of SH-SY5Y cells exposed to Aβ. Each point represents a distinct treatment condition: Aβ 2 μM alone (blue), Aβ + LYC at 1.6 μM (purple) or 8 μM (orange), and Aβ + GAL at 250 μM (orange) or 500 μM (purple).

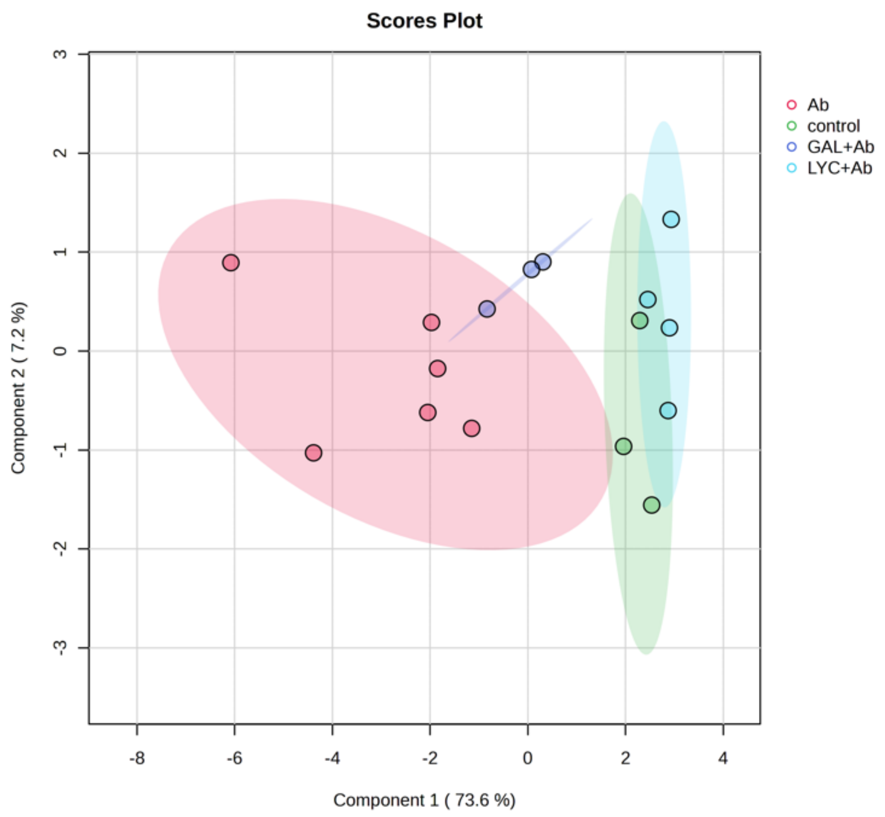

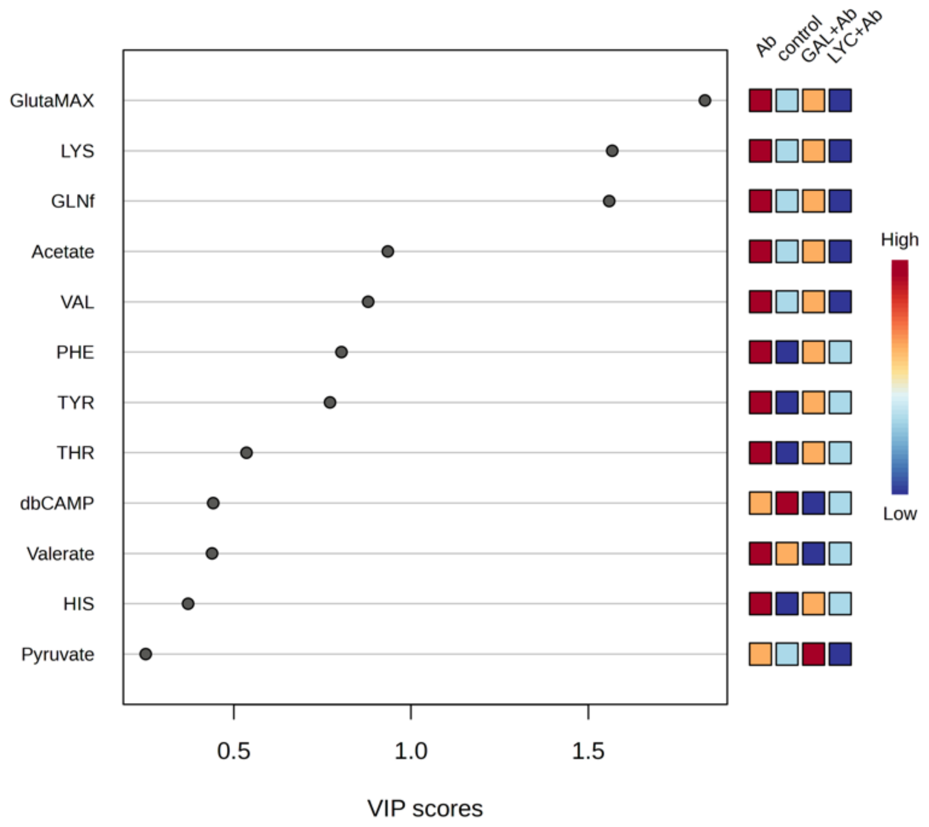

Figure S3. Partial Least Squares Discriminant Analysis (PLS-DA). The scores plot shows the distribution of different experimental groups: Aβ42 (red), control (green), GAL+ Aβ42 (blue), and LYC+ Aβ (light blue). The VIP scores plot (bottom) highlights the most relevant metabolites contributing to class differentiation.

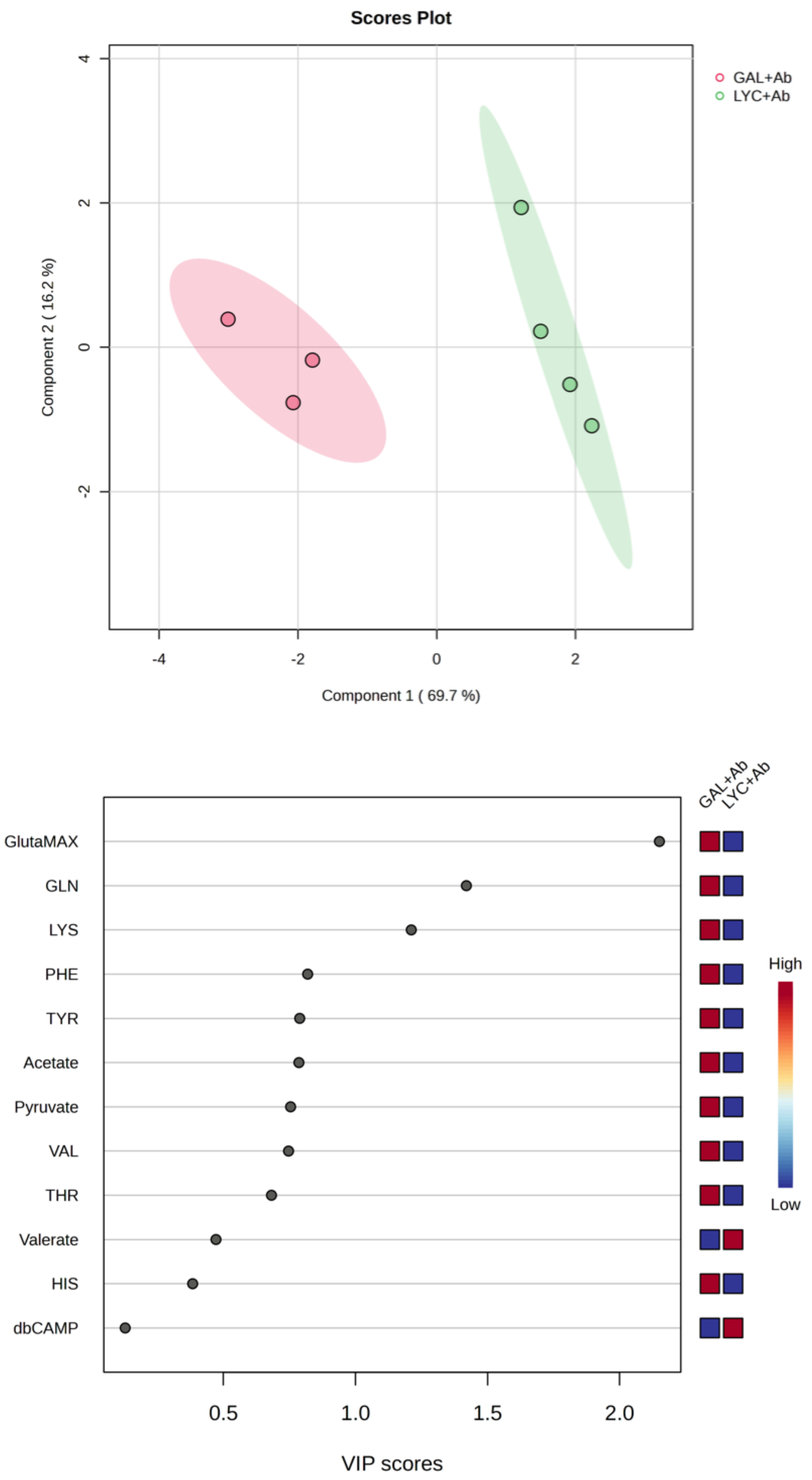

Figure S4. Partial Least Squares Discriminant Analysis (PLS-DA). The scores plot shows the distribution of different experimental groups: GAL+ Aβ42 (red), and LYC+ Aβ (green). The VIP scores plot (bottom) highlights the most relevant metabolites contributing to class differentiation.
